# Correlation Between Donepezil and QTc Prolongation and Torsades de Pointes: A Very Rare Phenomenon

**DOI:** 10.7759/cureus.6451

**Published:** 2019-12-23

**Authors:** Bilal Haider Malik, Pousette Hamid, Safeera Khan, Deepti Gupta, Muhammad Islam

**Affiliations:** 1 Internal Medicine, California Institute of Behavioral Neurosciences and Psychology, Fairfield, USA; 2 Neurology, California Institute of Behavioral Neurosciences and Psychology, Fairfield, USA; 3 Family Medicine, California Institute of Behavioral Neurosciences and Psychology, Fairfield, USA; 4 Reproductive Medicine, Saint Mary's Hospital, Manchester, GBR; 5 Pediatrics, California Institute of Behavioral Neurosciences and Psychology, Fairfield, USA

**Keywords:** dementia, donepezil, qtc prolongation, torsade’s de pointes, polymorphic ventricular tachycardia, syncope, falls, diarrhoea

## Abstract

Dementia can be seen as a clinical syndrome featuring a decline in cognitive and psychological abilities that can cause disability. Two major kinds of drugs are available: N-methyl-D-aspartic acid receptor antagonists like memantine and acetylcholinesterase inhibitors such as galantamine, rivastigmine and donepezil. In this article, we have reviewed the available literature along with the provision of a snapshot of published cases in the literature We used the PubMed database for our search. The average age of patients was 80 years and above. Patients described in the literature belonged to both female and male gender, with female patients being predominant. Patients demonstrated associated atrioventricular (AV) block or ventricular premature contractions (VPC) or atrial fibrillation (AF) prior to developing torsades de pointes (TdP). Presenting complaints were either syncope or diarrhoea or accidental bradycardia. Mostly, the corrected QT interval (QTc) normalisation was associated with discontinuation of donepezil. We recommend further studies to determine this correlation between donepezil and incidence of QTc prolongation and TdP.

## Introduction and background

Dementia can be seen as a clinical syndrome featuring a decline in cognitive and psychological abilities that can cause disability [1]. Alzheimer's disease (AD) is the most common type. Other types include mixed dementia, Lewy bodies dementia, and vascular dementia [1]. Dementia is now a worldwide problem. As projected, 135 million people will have dementia globally by 2050. The dementia care cost was calculated at $ 604bn (£ 396bn; € 548bn) in 2010 and is set to increase to $ 1tr by 2030 [2]. The assessment encompasses history, physical examination (including full neurological examination), cognitive evaluation, and baseline investigations (such as electrolytes and urea, erythrocyte sedimentation rate [ESR], full blood count [FBC], vitamin B12, folate, thyroid function tests [TFT]) [3]. Mini-mental state examination has been primarily suggested as the initial method for cognitive evaluation [4]. The Addenbrooke’s cognitive examination has a higher diagnostic yield than the mini-mental state examination but takes longer to administer [5-6]. Several contemporary dementia diagnostic criteria advocate the use of imaging for various dementias, including Lewy bodies dementia and AD [7-9]. Advances in imaging like single-photon emission computed tomography (SPECT) imaging is being used currently to ascertain the diagnosis of dementia with Lewy bodies [10]. With the recent advances in medicine, reliable dementia drugs have been produced whose focus is to enhance or retain function after neuronal injury instead of modifying the intrinsic aetiology [11]. There are two major kinds of drugs available (A) N-methyl-D-aspartic acid receptor antagonists like memantine and acetylcholinesterase (AChE) inhibitors like galantamine, rivastigmine, and donepezil [11]. Currently, the only suggested choices for managing mild-to-moderate AD are AChE inhibitors [12]. During one of the recent developments, findings from a large randomized controlled trial have shown that sustained treatment with donepezil in moderate to severe dementia is affiliated with improvement in cognition [13]. Memantine is authorized for use in patients with AD (moderate to severe) and AChE inhibitor intolerance, and its off-label use in mild AD has also been observed despite lack of evidence [14]. Cholinesterase inhibitors such as rivastigmine, donepezil, and galantamine are associated with cardiac conduction disorders [15]. We can find cases in the literature on corrected QT interval (QTc) prolongation and torsades de pointes (TdP) in patients who have been using donepezil for their cognitive decline [15].

## Review

Initially, we will be using a snapshot description of seven cases published/reported in the literature by other esteemed authors for the purposes of building a better understanding of this important topic.

Snapshot description of cases reported/published in the literature

Published Case-1

An 83-year-old female was admitted to the emergency department (ED) after a syncopal episode and hip pain. She was diagnosed with a fracture neck of femur and had a past medical history of recurrent falls, Alzheimer’s dementia, and hypertension. Medications included 10 mg of donepezil, 2.5 mg of bendroflumethiazide, and 20 mg of simvastatin. Electrocardiogram (ECG) showed prolonged QTc of 638 milliseconds. All blood tests were normal, and no postural drop of BP was noted. Falls were attributed to cardiogenic syncope in view of prolonged QTc secondary to donepezil, which was subsequently withheld. Repeat ECG at days 2 and 10 demonstrated normal QTc (436 milliseconds). Follow-up outpatient 24-hour ECG and echocardiogram were also normal. The patient was discharged from the hospital after input from the orthopaedics team for fracture and MDT [16].

Published Case-2

An 80-year-old female presented to the ED with a couple of days history of diarrhoea and anorexia and a past medical history of atrial fibrillation (AF), pacemaker in situ, hypertension, cerebrovascular disease, and mixed vascular/Alzheimer’s dementia. Medications included 8 mg of perindopril, 2 mg of bumetanide, 20 mg of atorvastatin, 10 mg of donepezil (increased from 5 mg to 10 mg two weeks ago with no prior ECG), 60 mg of fluoxetine, 60 mg of diltiazem M/R, and 30 mg of lansoprazole Admission ECG showed QTc prolongation. During admission, she developed polymorphic ventricular tachycardia (TdP) with unconsciousness. Cardiac output was regained after CPR and 200J shock. Donepezil was withheld and QTc normalised [17].

Published Case-3

An 86-year-old female was admitted to the hospital because of a syncopal attack. Her past medical history included AD and hypertension. Medications included 5 mg of amlodipine and 5 mg of donepezil. Her general physical and targeted systems examination including initial observations were also normal. Initial investigations including the majority of cardiovascular workup were normal except the ECG which showed AF with normal QTc. She was kept under observation with ECG monitoring. On the second day, ECG monitor picked up TdP for 10 seconds which terminated spontaneously. Subsequent ECG showed normal sinus rhythm with normal QTc. Intravenous lidocaine infusion was commenced. For the next two hours, ECG monitor picked up TdP five times, all terminating spontaneously with 30 seconds. The patient felt no symptoms. Donepezil was withheld. Subsequently, coronary arteriogram and catheter examination along with programmed ventricular stimulation were performed, which all concluded to be normal. The patient was commenced on bisoprolol and metildigoxin for paroxysmal AF (PAF) with medical outpatient follow-up. The patient was discharged home without donepezil with normal discharge ECG (normal QTc) [18].

Published Case-4

An 80-year-old female with AD was commenced on donepezil. She was also known to be taking 8 mg of benidipine and 10 mg of atorvastatin that could potentially interact with donepezil. In the community, the patient was reviewed every month with an ECG. First to third ECG demonstrated prolonged QTc (average 470+/-9). In view of her risk factors and risk of developing TdP, her physician was informed and her benidipine was switched with amlodipine, which produced a decrease in QTc from fourth to seventh ECG (average 441+/-9 milliseconds), demonstrating a reduced risk of TdP [19].

Published Case-5

An 83-year old woman presented with diarrhoea, vomiting, and syncope. Her past medical history was hypertension, PAF, MI, and diabetes. Her medications included 5 mg of donepezil and 5 mg of bisoprolol. A working diagnosis of acute colitis was made. Initial investigations revealed raised white blood cell (WBC) count, low potassium, and high brain natriuretic peptide (BNP). ECG demonstrated Ischaemic changes with prolonged QTc of 645 milliseconds [20]. Chest X-rays (CXR) showed cardiomegaly. Echocardiography revealed aneurysmal apex. Frequent ventricular premature contractions were observed on ECG monitoring. Fluid resuscitation with supplemental potassium was commenced. After a few hours, TdP was detected on ECG monitoring lasting 35 seconds. IV magnesium and lidocaine were commenced; however, she developed another episode of TdP but this time with syncope and transient convulsions. She was commenced on isoprenaline which decreased the frequency of VPCs, and TdP was not detected again. Coronary angiogram revealed triple vessel disease. After the discontinuation of donepezil QTc decreased to 485 milliseconds prior to discharge [20].

Published Case-6

A 90-year old male was admitted to the hospital with accidental bradycardia. Past medical history included AD. Patient’s medication included 10 mg of donepezil. The patient was asymptomatic with initial vitals recording bradycardia at 36/min. Initial physical examination and laboratory tests were unremarkable. ECG showed 2:1 advanced atrioventricular block with signs of ischaemia and prolonged QT at 0.514 seconds. In view of this, donepezil was stopped and orciprenaline was commenced. Subsequent ECGs showed sinus rhythm with first-degree block and QTc prolonged at 0.538 seconds. On the fifth day, ECG showed QTc reduced to 0.456 seconds [21].

Published Case-7

An 87-year-old female was admitted to the hospital because of syncope and subsequent fall sustaining a vertebral fracture. Her past medical history was AD, bradycardia, AF, and hypertension. Her medications included 5 mg of donepezil, 1 mg of warfarin, 25 mg of spironolactone, 5 mg of amlodipine, and 100 mg of cilostazol. Initial investigations and assessment, except heart rate (HR) and ECG were unremarkable [21]. Her HR was 40/min with ECG, demonstrating AF with QTc of 0.594 seconds [21]. Few hours into hospitalisation, the patient developed nausea and vomiting with ECG monitoring, demonstrating TdP followed by R on T developing ventricular fibrillation. This rhythm reverted back to original rhythm after about three minutes. Donepezil was discontinued. Following discontinuation of donepezil, QTc gradually reduced to 0.446 seconds on the 18th day [21].

Table 1 below summarises the aforementioned published cases.

**Table 1:** Snapshot description of published cases in the literature [16-21]

Case No	Reporting Year	Country	Presenting Complaint	QTc Prologation	Torsade's de Pointes	Donepezil Discontinued	QTc Reduced Post Intervention	Author
1	2019	UK	Syncope	Yes	Not Documented	Yes	Yes	Jackson EG et al.
2	2015	UK	Diarrhoea & anorexia	Yes	Yes	Yes	Yes	Kitt J, et al.
3	2013	Japan	Syncope	No	Yes	Yes	Yes	Hadano Y, et al.
4	2012	Japan	Prolonged QTc	Yes	No	No	Yes	Kohki Shinozaki, et al.
5	2009	Japan	Diarrhoea, vomiting, and syncope	Yes	Yes	Yes	Yes	Takaya, Tomofumi, et al.
6	2009	Japan	Accidental bradycardia	Yes	No	Yes	Yes	Atsushi Tanaka, et al.
7	2009	Japan	Syncope & fall	Yes	Yes	Yes	Yes	Atsushi Tanaka, et al.

Dementia and CNS cholinergic system

Dementia symptoms are thought to be associated with abnormal neurotransmission and degradation of neuronal circuits in the affected areas of the brain [22]. Cognitive impairment in patients with likely AD is correlated with a gradual loss of cholinergic neurons and a resultant decline in brain levels of acetylcholine (ACh), notably hippocampus, temporal and parietal lobe [23-24]. There is a cholinergic deficit in the brains of patients with Lewy bodies dementia, AD and vascular dementia [25-27]. Cholinergic replacement therapy could possibly help all patients with dementia as findings indicate that abnormality of cholinergic function leads to the symptoms in all three forms of dementia [28]. Two cholinesterases, butyrylcholinesterase (BuChE) and AChE, destroy ACh in the brain [29]. While AChE is noticed to be in higher concentrations than BuChE in the brain of AD patients, there is proof to suggest that BuChE is active in cortical and hippocampal areas with cholinergic innervation [29]. Changes related to cholinergic denervation are associated with the appearance of neurofibrillary tangles and amyloid plaques in the brains of patients with AD [30]. In vascular dementia, cognitive impairment is associated with brain hypoperfusion or ischemia, commonly seen in association with cardio/cerebrovascular disease [31]. Lewy bodies, comprising abnormal neurofilament proteins, manifest in the limbic area of LBD patient's brain and are indicators for neuronal deficit [32].

Acetylcholinesterase Inhibitors

AD patients who are given cholinesterase inhibitors attain symptomatic improvements as these drugs increase the concentration of ACh in brain’s neuronal synapses by stopping the breakdown of ACh by cholinesterase thereby augmenting the cholinergic processes [28]. While numerous strategies have been evaluated to enhance cognition and cholinergic functions in AD patients, cholinesterase inactivation has stood out as the only approach to benefit the patients [28]. Since the pharmacological and pharmacokinetic characteristics of the most commonly utilized cholinesterase inhibitors have major differences that may impact their efficacy, the importance of these stays theoretical in view of the lack of large, randomized control studies that compare and contrast different cholinesterase inhibitors with one another [28]. Increasing evidence suggests that BuChE and AChE play a vital role in cholinergic transmission [33-34]. For this purpose, when dealing with AD, both cholinesterases are seen as valid goals of treatment [33-34]. Donepezil and galantamine both selectively inhibit AChE, while both BuChE and AChE are inhibited by rivastigmine [35].

Acetylcholinesterase inhibitors: cardiac conduction disorder (QTc prolongation and TdP)

**Figure 1:**
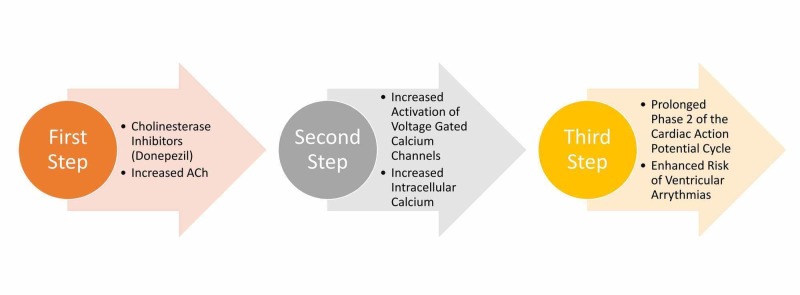
Proposed mechanism of ventricular arrhythmias with donepezil

Galantamine, rivastigmine, and donepezil have shown to be associated with a higher incidence of gastrointestinal (GI) side effects. Cardiovascular system (CVS) side effects include bradycardia. Donepezil has been reported to be associated with syncopal episodes [28]. Other common side effects include muscle cramps and insomnia [28]. As described above, the association of donepezil with syncopal episodes is assumed to be due to a few reasons, being described as concomitant ischemic heart disease (IHD) and low potassium levels in addition to bradycardia [20]. It can also be attributed to the development of arrhythmias like TdP mostly associated with prolonged QTc [20]. As per cardiac physiological studies, voltage-gated calcium channels open as a result of the activation of cardiac ACh receptors. One of the logical conclusions of AChE inhibition will be the increased level of calcium within the intracellular space. Increased intracellular calcium levels can lead to prolongation of phase 2 of the cardiac action potential cycle, which in turn can lead to an enhanced risk of ventricular arrhythmias [18]. Figure 1 describes the proposed mechanism of donepezil leading to ventricular arrhythmias.

It is also important to note some of the risk factors identified to act synergistically in this scenario, which are IHD, electrolyte disturbances (hypocalcaemia, hypokalaemia or hypomagnesaemia), female sex, old age, congestive heart failure, atrial flutter, AF, bradycardia, drug overdoses, rapid IV drug administration, polymorphism of genes coding ion channels or enzymes involved in drug metabolism, use of drugs that prolong the QTc, congenital long QT syndrome, and family history of sudden death [18]. In some of the case reports described in the literature, an association has been found between donepezil and ventricular arrhythmias, especially TdP, QTc prolongation, bradycardia, and heart blocks [17]. Other precipitating factors identified to be associated with the above-mentioned cardiac conduction disorders from these case reports are hypokalaemia, acute colitis, diarrhoea, apical cardiac aneurysm, triple vessel disease, previous MI, and interactions with drugs like fluoxetine and benidipine (beware of drugs that prolong QTc) [17].

## Conclusions

Our review article points towards a possible correlation between donepezil and the incidence of QTc prolongation and the subsequent development of torsades de pointes in the elderly population with dementia. We think it remains to be ascertained whether this very rare phenomenon/side effect related to donepezil use is due to donepezil itself or a mere incidence of chance in these cases. We recommend further studies to determine this correlation between donepezil and the incidence of QTc prolongation and TdP. In the meanwhile, we advise physicians prescribing donepezil to remain vigilant about the incidence of QTc prolongation and TdP as a very rare side effect of donepezil.
